# Who Shall Not Be Treated: Public Attitudes on Setting Health Care Priorities by Person-Based Criteria in 28 Nations

**DOI:** 10.1371/journal.pone.0157018

**Published:** 2016-06-09

**Authors:** Jana Rogge, Bernhard Kittel

**Affiliations:** 1 Department of Special Education and Rehabilitation, University Oldenburg, Oldenburg, Germany; 2 Department of Economic Sociology, University of Vienna, Vienna, Austria; Centre Hospitalier Universitaire Vaudois, FRANCE

## Abstract

The principle of distributing health care according to medical need is being challenged by increasing costs. As a result, many countries have initiated a debate on the introduction of explicit priority regulations based on medical, economic and person-based criteria, or have already established such regulations. Previous research on individual attitudes towards setting health care priorities based on medical and economic criteria has revealed consistent results, whereas studies on the use of person-based criteria have generated controversial findings. This paper examines citizens’ attitudes towards three person-based priority criteria, patients’ smoking habits, age and being the parent of a young child. Using data from the ISSP Health Module (2011) in 28 countries, logistic regression analysis demonstrates that self-interest as well as socio-demographic predictors significantly influence respondents’ attitudes towards the use of person-based criteria for health care prioritization. This study contributes to resolving the controversial findings on person-based criteria by using a larger country sample and by controlling for country-level differences with fixed effects models.

## Introduction

An aging population and progress in medical technology generate upward pressure on health care expenditures. It has repeatedly been argued that the distribution of these resources based purely on medical need is undermined by increasing mismatch of demand and supply [1, 2]. The rapid development of new health care technologies leads to annual increases in government health expenditure between two and three per cent [3, 4]. Forecasts indicate that the future costs of medical services may be insufficiently covered by increased social insurance contributions or taxes [5]. If this prediction holds true, the question may not be whether or not explicit priority regulations must be introduced, but which criteria should be used in setting priorities [6–8].

The limiting of health care resources affects citizens who contribute to the funding of the public health care system. Policy makers are therefore well advised to take citizen preferences into account when defining explicit rules of prioritization [9–10].

Commentators argue that defining priority criteria raises the possibility of allocating scarce and inseparable health resources in a fair and transparent process [2, 11]. Numerous studies have shown that individuals are more willing to accept collective decisions against their interest if they result from a fair process [12–16].

Criteria for setting explicit priorities can be divided into three groups: (i) medical (severity of the disease, treatment required, etc.); (ii) economic (treatment costs, cost-effectiveness ratio, etc.); (iii) person based (health-related lifestyle, age, social responsibility, etc.) [17]. While several empirical studies have shown that the vast majority of respondents support medical criteria [18–27] and reject economic criteria [19, 22, 24, 27, 28], the findings about individual attitudes towards person-based criteria are inconsistent and vary according to surveyed country, selected operationalization and research design [29].

Explicit priority experiences in several countries (Israel, United Kingdom, New Zealand, and the United States (Oregon)) show that rationing purely based on medical criteria does not compensate for the financial problems [7, 30, 31]. The annual increase in health care expenditures by far exceeds savings made by priority setting.

Since numerous empirical studies have already concluded that economic criteria are not acceptable to citizens, the purpose of the present study is to analyze citizens’ attitudes towards subordinated person-based criteria in a cross-country sample.

### Theoretical framework and empirical findings

An ethical argument for using a patient’s age as a criterion for priority setting could be based either on efficiency [32] or fair-innings considerations [33]. While the latter is implied by the idea that older patients have already experienced a longer life and that younger patients should be given the chance to achieve the same [34], the efficiency argument claims that curing younger people results in longer benefit duration after treatment [20, 35].

However, several arguments have been raised against the use of age. Firstly, while the treatment of elderly patients is costly, it may still help to reduce the costs to society as a whole [36]. For example, surgery on an elderly patient may be less costly than the expenditures that might be incurred in the case of no treatment. Secondly, prioritizing according to age ignores the societal contributions of older people, such as retired people taking care of young or old family members [37]. Thirdly, using age as a priority principle might also contribute to the reproduction of social inequalities. Whereas older people with higher socio-economic status often purchase private supplementary insurance, this is not possible for poorer older people [37].

Taking patients’ health-related lifestyles into account refers to the principle of personal responsibility [37]. Proponents of liberal egalitarianism argue that society should only pay for health expenditures whenever the need for a treatment can be attributed to factors that are beyond personal control. From this perspective, illnesses caused by individual behavior should be proportionally or fully financed by additional individual taxes or health insurance contributions of those who made these choices [9].

This view has also been subject to various criticisms. The first challenge of using a patient’s health related lifestyle as a priority criterion is the problem of accountability. In the case of a smoker diagnosed with lung cancer the causal mechanism is rather obvious. However, should smokers also be made responsible for diseases that are not clearly linked to their smoking habit? Second, other factors such as social background or the level of education are often prior to behavior and thus using a patient’s lifestyle as a criterion might reproduce social inequalities [9, 19]. Lastly, there is no common standard for assessing different health-damaging behaviors, raising new problems of fairness and justice [38, 39].

A second argument that has been forwarded in favor of taking patients’ health-related lifestyle into account is based on an efficiency consideration. Patients with a healthy lifestyle are expected to uphold this lifestyle after treatment, whereas people living unhealthily before treatment are more likely to relapse after medical treatment [35, 37]. The expected benefit is therefore greater for patients with a healthy lifestyle in the first place.

The proposal to consider a person’s social responsibility in terms of obligations to care for others, such as a young child or other relatives, is grounded on an efficiency argument. If, from this perspective, curing a person is less costly than publicly substituting his or her care responsibilities, the society is better off by providing the treatment [37, 38].

Recent studies on attitudes towards person-based priority criteria have analyzed whether a patient’s age, health-related lifestyle, and social responsibility should be considered for prioritization decisions. Given that the framing of the study significantly affects the acceptance of a criterion [37], we classify those studies into five categories.

The first category contains survey studies in which support for a criterion is measured by an item stating a demand for the general exclusion of specific groups of people from public health care. These studies conclude that the vast majority of respondents do not support the idea that older people should be excluded from access to public health care [40–42]. In contrast, analyses using this framing to capture attitudes towards the overall exclusion of patients with an unhealthy lifestyle are inconsistent. For example, while only a very small number (1 per cent) of British focus group participants support the idea that smokers should have access to public health care, the majority of Finnish responding to a survey endorse such a regulation [43–45]. However, although both studies have used patients’ smoking habits as an indicator for their health related lifestyle, the differences may be a result of distinct country-specific samples or artifacts of different methods.

The second category measures support for a criterion using the respondent’s general agreement to an overall prioritization of the higher priority group. Studies on attitudes towards the idea that younger patients should generally be prioritized over older patients show that only a minority accepts this criterion [19, 20, 22, 28]. Analyses that used this framing to investigate attitudes towards health-related lifestyle [22, 27, 44, 45] or social responsibility [27, 28] have reached similar conclusions.

A third category of studies focuses on the support for a criterion in comparison to other possible criteria, such as, for example, medical needs. All studies investigating the relevance of person-based criteria in relation to medical criteria conclude that, for the vast majority of respondents, personal characteristics are subordinate to medical criteria [20, 21, 24, 40, 46].

The fourth category consists of studies that measure support for a criterion by the level of approval it enjoys within prioritization decisions. This framing has only been used to investigate attitudes towards the use of patients’ health-related lifestyle. While in a Finnish survey the majority (66 per cent) of participants accept the consideration of health-related lifestyle to set priorities in any form, a German study concludes that only 49 per cent of Germans support this criterion for treatment decisions. Both studies use a representative national survey [19, 42]. The different findings thus may indicate differences between countries. Alongside these two representative studies, an interactive student survey reveals that the vast majority (90 per cent) of surveyed German students support the idea of taking patients’ lifestyles into account [46], in contrast to only 25 per cent of participants in a Swedish survey [25].

The last category includes studies in which respondents were confronted with a zero-sum scenario of two patients in need of the same treatment who only differ in one of the personal criteria, and where only one patient can receive treatment. Respondents were asked to prioritize one patient over the other. All studies indicate that the vast majority accepts the prioritization of younger over older patients [47–49] and patients with a healthy lifestyle over risk-taking patients [28, 47].

In conclusion, a clear majority supports the consideration of patients’ age or health-related lifestyle if two patients, who only differ in age or lifestyle, compete for limited health care resources. This hypothesis is supported by findings from studies that analyze the relevance of a person-based criterion in relation to other possible criteria. In contrast, the majority of respondents clearly reject a general prioritization of younger patients as well as the overall prioritization of patients with a healthy lifestyle or with social responsibilities. Furthermore, the majority of respondents disagree with the idea of a general exclusion of older people or patients with an unhealthy lifestyle from the public health care system.

Findings for individual factors associated with citizens’ attitudes towards age, health-related lifestyle and social responsibility are rare and partly inconsistent because of the diversity of framings. Since all priority criteria are based on at least one distributive principle, citizens’ attitudes towards these criteria should be directly correlated with distributive preferences. Therefore, prioritization preferences are expected to vary with regard to the same factors as distributive preferences [29]. According to this hypothesis, attitudes towards the use of person-based priority criteria should be affected by self-interest as well as by the individual’s socialization [50].

To the extent that attitudes are driven by self-interest, respondents’ support for person-based criteria should depend on the implications of a prioritization criterion for themselves [51]. Hence, smokers would be expected to be significantly less likely to support a criterion related to patients’ smoking habits, the elderly would be less willing to accept age as a criterion, and respondents without a young child would more often reject the idea of prioritizing patients with young children. In line with this hypothesis, an Australian study concluded that smokers more often reject a patient’s lifestyle as a criterion to set health care priorities [28]. Moreover, numerous studies have shown that support for age prioritization is negatively related to the respondent’s own age [20, 21, 25, 42]. However, other studies have found a positive correlation between age and support of age as a criterion [28, 47].

According to socialization theory people adopt specific norms and values in childhood and adolescence which determine their attitudes, beliefs and skills [52]. Socialization varies with social variables such as age, gender, social position, or educational achievement. These factors have been shown to affect distributive preferences [50, 53–55]. One can thus expect that people in lower social positions reject a prioritization based on personal characteristics more often than respondents in a more favorable economic position. However, none of these studies have found a correlation between social position and attitudes.

Gender-specific differences in distributive preferences are based on the assumption that women depend more frequently on social welfare than men and are therefore more in favor of resource allocations based on need and equality [54]. Additionally, women and men differ with regard to the role expectations experienced during socialization [53]. While women are typically expected to develop social-emotional skills, men have to acquire achievement-oriented behaviors to fulfill the requirements made on them [56]. In line with those expectations, an American telephone survey indicates that men are more in favor of age-based priority setting than women [49]. However, in a Swedish survey women supported age as a criterion for defining priorities more often than men [50]. Studies on the support of health-related lifestyle and social responsibility criteria found no significant differences with regard to the respondents’ sex.

Age related differences with regard to distributive preferences are expected to result from the effect of aging. It is assumed that older people tend to attach more causal importance to individual factors for personal living conditions [56, 57]. Older people should thus be more in favor of criteria emphasizing the individual responsibly of patients than younger respondents. Evidence from an Australian and a Finnish survey suggest that age is positively correlated with support for a patient’s health related lifestyle as a priority criterion [19, 28].

A similar argument is used to explain divergent preferences between people with different levels of education. The more educated are more likely to adhere to the view that individual performance should pay off [50]. However, none of the studies that analyzed attitudes towards the use of a patient’s health-related lifestyle found a relationship between the respondent’s level of education and support for this criterion. On the other hand, the evidence seems to indicate that people with a university background are more in favor of age-based priority setting than less educated people [49]. This finding might indicate that efficiency considerations play a greater role for more educated people. We are not aware of studies regarding individual factors associated with attitudes towards parents of a young child.

To sum up, most of the studies analyzing attitudes towards the use of person-based criteria have been administered through a national sample using different framings. Comparing these findings is therefore difficult. The same holds for studies on individual factors that influence peoples’ attitudes towards the use of person-based priority criteria. The purpose of this study is thus to examine the extent to which respondents in 28 nations support the use of the three most frequently studied person-based criteria, that is, patients’ lifestyles, age, and social responsibility, as subordinated prioritization criteria in situations where a choice needs to be made. Since attitudes towards priority criteria are expected to directly correlate with distributive preferences [29], the study will also examine whether or not attitudes towards the criteria vary according to a respondent’s self-interest and individual characteristics.

## Methods

### Sample

The empirical analysis of this paper is based on data from the ISSP Health Module (2011). All data are publicly available at ISSP Research Group (2015): International Social Survey Programme: Health and Health Care—ISSP 2011. GESIS Datenarchiv, Köln. ZA5800.

Among other topics, the dataset features the respondents’ attitudes towards the allocation of health care resources. The survey has been administered in 28 countries (Denmark, Croatia, Bulgaria, Norway, Sweden, Slovenia, Lithonia, Poland, Finland, The Netherlands, Switzerland, Germany, Japan, Belgium, Australia, Czech Republic, The United States, France, Great Britain, Chile, South Korea, South Africa, Israel, Russia, Turkey, Slovakia and Portugal) with a total sample size of 43,364 respondents. Sampling procedures differ across the participating countries [58].

Since the number of cases decreases by about 17,000 once all cases with at least one missing value in a predictor or dependent variable are excluded, we have used multiple imputation by chained equations (MICE). Given that using imputed values for the dependent variable would just add noise to the estimation, we only used imputed values for the independent variables and excluded all cases with missing values on at least one dependent variable [59, 60]. This resulted in a total net sample size of 36,199 respondents from 28 countries. The resulting sample composition is described in Table 1.

**Table 1:** Sample characteristics.

	Number of respondents	Proportion in %
**Gender**		
Male	16,377	45.24
Female	19,822	54.76
**Social position**		
In paid work	19,554	53.93
Unemployed	2,162	5.97
In education	1,931	5.33
Apprentice/Trainee	162	0.42
Disabled	1,243	3.43
Retired	7,443	20.56
Others	3,382	9.34
**Education**		
Low education	12,493	34.51
Medium education	14,062	38.85
High education	9,255	25.57
**Young child in household**		
Yes	6,463	17.85
No	29,736	82.15
**Smoking habit**		
Smoker	8,430	23.31
Non-smoker	27,772	76.72
**Aged 70 or older**		
Yes	4,062	11.22
No	32,137	88.78
**Total**	36,199	100

Multiple Imputation (m = 5)

### Compliance with Ethical Standards

The International Social Survey Programme is a collaborative large-scale project with representatives from all over the world. The aim of this collaboration is to collect high quality comparative research data on issues of general sociopolitical interest. The ISSP source questionnaires are developed and pretested by international teams and discussed and approved by the ISSP General Assembly (GA), which is the main representative body of the ISSP. The GA approves questions based on their scientific merit, sociopolitical relevance and ethical appropriateness. ISSP members, the national field questionnaires and field work must all comply with the given legal requirements in each country. Before depositing data to the ISSP Archive national ISSP, data are anonymized so that individual survey participants cannot be identified [61].

### Data Analysis

The statistical analysis comprises two stages. First, we present descriptive statistics on the proportion of respondents who support the use of the three person-based criteria and explore differences in these proportions across the 28 countries. Second, we statistically test the effect of socio-demographic (gender, age, income, social position, and level of education) and behavioral characteristics (individual health behavior) on attitudes towards the use of patients’ smoking habits, age and parental status as a prioritization criterion. Given that the hypotheses are framed as universal claims, we employ a fixed-effects logistic regression model, which controls for differences in intercept between countries but assumes common coefficients across countries. All data analyses are carried out with Stata SE 13.

### Measures

#### Attitudes towards person-based priority criteria

Respondents’ attitudes towards the use of person-based priority criteria were measured by using a scenario where patients are competing for limited health care resources. The ISSP Health Module (2011) includes three items on respondents’ attitudes towards the use of person-based priority criteria for health care, one for each of the three criteria. Age is measured in years, a patients’ lifestyle is operationalized by smoking habits, and social responsibility is represented by a patient being the parent of a young child. Respondents were asked to decide which of two equally sick patients should be scheduled for heart surgery first, given that (i) one is 30 years old and the other is 70 years old, (ii) one is a heavy smoker and the other is a non-smoker, and (iii) one has a young child and the other has not. The respondents were then asked to choose between three response categories. The first category implies support for the person-based criteria in favor of the younger, the non-smoker, or the patient with a young child. The second category stands for prioritizing the smoker, the older patients or the patient without a young child. The third category implies the rejection of the idea of person-based prioritization. Because this study focuses on attitudes towards the prioritization of younger over older people, non-smokers over smokers, and patients with young children over patients without young children, and since the response to the second category is very low (1.7–4.1 per cent), we created a dummy variable for each criterion, each contrasting the first and the third category. The second category was excluded from the analysis. We coded respondents who chose the first category as supporters of the priority criterion “1”, and respondents choosing the third category “0”. Since all dependent variables are binary, logistic regression models were used.

#### Self-interest

All variables used to examine the influence of self-interest on attitudes towards the use of person-based priority criteria are dichotomous. All respondents smoking at least one cigarette a day are coded as “smokers”. Respondents who have at least one young child in their household are coded as “being the parent of a young child”. All respondents age 70 or above are coded as “age 70 or older”.

#### Individual background

Gender is dichotomous (1 = female; 0 = male). Social position is measured by seven dummy codes representing different types of work status (unemployed; in school; in training/student; permanently sick/disabled; retired; other; in paid work (for the reference group)). The level of education is measured by three dummy variables: low education (no school education or primary school); intermediate education (secondary school or high school graduation); high education (university degree). Age in years is measured as a continuous variable. In order to make household incomes more readily comparable, we have converted currencies to international dollars based on data provided by the World Bank 2011 [62].

## Results

Table 2 shows the overall proportions of support for the three person-based priority criteria. The range of support varies between almost 40 per cent for the prioritization of patients with a young child and 46 per cent for age. Although none of the three criteria is supported by the majority of respondents, a considerable minority seems to accept the use of patients’ smoking habits, age and parental status as criteria to decide who shall be treated if a choice needs to be made.

**Table 2:** Overall proportion of support.

	Support		Reject	
	(N)	(%)	(N)	(%)
**Smoking habit**	15,318	42.32	20,881	57.68
**Age**	16,668	46.05	19,531	53.95
**Parental status**	14,384	39.74	21,815	60.26

N = 36,199

**Fig 1:**
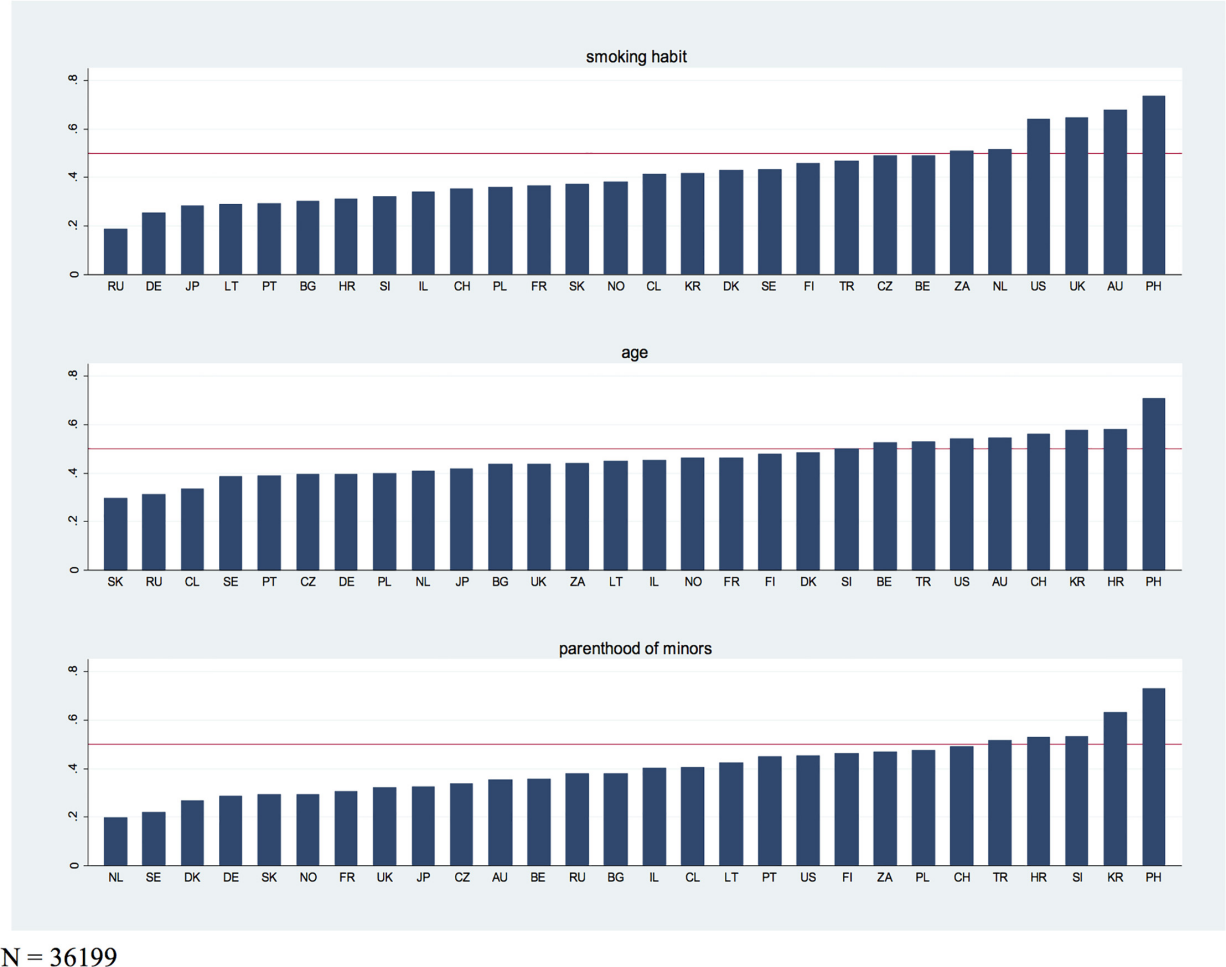
Proportion of support for prioritization by smoking habit, age and parental status as subordinated priority criteria by country.

Fig 1 presents the proportion of support for the three criteria in each of the surveyed countries. The graph at the top shows that a majority in six countries (South Africa, The Netherlands, The United States, United Kingdom, Australia, Philippines) supports patients’ smoking habits as a criterion to prioritize.

While the highest level of support for smoking habit as a prioritization criterion is observed in the Philippines (78 per cent), the lowest level of support is observed in Russia (just under 19 per cent). The graph in the middle displays the between-country variation in the support for age as a prioritization criterion, which is rather small as compared to the two other criteria. The highest level of support is again observed in the Philippines (71 per cent). A majority agreeing to this criterion can also be observed for Slovakia, Belgium, Turkey, The United States, Australia, Switzerland, South Korea, and Croatia. As shown in the bottom graph, being the parent of a young child is considered a prioritization criterion by a majority in five countries. Once more, support is highest in the Philippines (73 per cent) while it is lowest in The Netherlands (19 per cent).

Table 3 presents the results of statistical tests of the effect of self-interest and individual characteristics on attitudes towards the use of person based priority criteria. All country heterogeneity in intercepts is absorbed by the inclusion of N-1 country dummies [63].

**Table 3:** Fixed-Effect logistic regression models: influencing factors on peoples’ support of smoking habits, age and parental status as priority criteria.

	M1: Support for smoking habits	M2: Support for age	M3: Support for parental status
Smoker	0.372***		
	[0.351,0.395]		
Older than 70		1.288***	
		[1.190,1.394]	
Young child in household			1.292***
			[1.213,1.376]
Age in years	1.000	-	1.009***
	[0.998,1.002]		[1.007,1.011]
Female	0.939**	0.921***	0.894***
	[0.897,0.983]	[0.882,0.962]	[0.855,0.936]
Level of education (ref. high)			
Low	0.965	1.030	1.228***
	[0.906,1.027]	[0.970,1.093]	[1.154,1.308]
Intermediate	0.979	1.029	1.146***
	[0.924,1.037]	[0.974,1.087]	[1.082,1.214]
Social position (ref. in paid work)			
Unemployed	0.929	1.097	1.216***
	[0.840,1.026]	[1.000,1.205]	[1.105,1.338]
In school	1.184**	1.220***	1.243***
	[1.064,1.317]	[1.109,1.342]	[1.115,1.386]
In training/student	1.346	1.308	1.361
	[0.969,1.869]	[0.956,1.790]	[0.974,1.900]
Permanently sick/disabled	0.969	0.964	1.225**
	[0.850,1.106]	[0.853,1.090]	[1.078,1.392]
Retired	1.011	0.868***	1.245***
	[0.937,1.091]	[0.813,0.927]	[1.155,1.342]
Others	1.083	1.158***	1.369***
	[0.996,1.177]	[1.069,1.255]	[1.260,1.488]
Household income in 1000 international $	1.000	1.000	1.000
	[1.000,1.000]	[1.000,1.000]	[1.000,1.000]
Country Dummies	Yes	Yes	Yes
*N*	36,199	36,199	36,199
Average adjusted R^2^	0.072	0.023	0.050
Average adjusted R^2^ fixed effects only	0.045	0.021	0.039

Coefficients are Odds Ratios; 95% confidence intervals in brackets

* p < 0.05

** p < 0.01

*** p < 0.001; Multiple Imputation (m = 5)

As reported in the bottom row of Table 3, the proportion of variance explained by country heterogeneity varies between 2.1 per cent for age and 4.5 per cent for smoking habit. Therefore, in line with expectations, most differences with regard to attitudes towards the use of person-based priority criteria are to be explained by variation in the individual characteristics of respondents. Hence, in a second stage individual-level variables are added in order to test for the effect of individual characteristics on attitudes towards the use of person based priority criteria.

Because the relationship between the dependent and the independent variables is nonlinear in logistic regression, we cannot directly interpret the coefficients (Logits). Therefore, we displayed the effects as Odds Ratios instead of Logits. Although Odds Rations are a useful alternative to compare the effects of different independent variables on the dependent variable, they are also difficult to interpret. We thus converted the Logits (S1 Table) into predicted probabilities and calculated these for selected values of significant predictors [64].

Turning first to self-interest, observable effects are clearly in line with expectations for the support of patients’ smoking habits (Model 1). Setting all other factors to their mean, the probability for support changes by 22 percentage points between smokers (25 per cent) and non-smokers (47 per cent) (p < 0.001). Gender also appears to be a relevant, though much less powerful, predictor. While the probability of support is 40.7 per cent for women, it increases by 1.6 percent points for men (p < 0.01). With regard to the respondents’ social position, the only significant difference occurs between respondents in education and respondents in paid work. While the probability of support is 45.3 per cent for the former, it decreases by 4.1 percentage points for the latter (p < 0.01). Note that neither age nor the level of education seem to have a significant impact on attitudes towards patients’ smoking habits as a priority criterion.

In sum, the three relevant individual predictors explain an additional 2.8 per cent of the variance in attitudes towards the use of patients' smoking habits as a priority criterion. Additionally, among these factors, the respondents’ own smoking habit, and therefore their self-interest, seems to be the strongest predictor.

Model 2 presents the results of the model for the acceptance of age as a priority criterion. Respondents aged 70 or older do not reject prioritization by age more often than younger respondents. It rather appears that older respondents are more in favor of using age as a priority criterion (p < 0.001). Therefore, the findings are not in line with the self-interest hypothesis. The probability of support is 6 percentage points higher for respondents aged 70 plus. Like before, women seem to reject the use of the age criterion significantly more often than men (p < 0.001). With all other factors set to their mean, the probability for support is 2 percentage points higher for male than for female respondents. Additionally, the analysis shows that respondents who are unemployed (p < 0.05), in education (p < 0.001), or other (p < 0.01), are much more in favor of age as a priority criterion than respondents in paid work. In contrast, the probability for support is 3.5 percentage points higher for respondents in paid work (46 per cent) than for retired respondents (42.5 per cent). The error probability for this effect is less than 1 per cent. While age, gender and social position all seem to have a significant influence on attitudes towards age-based priority setting, the variance explained by these factors is very low (0.2 per cent).

With regard to attitudes towards the prioritization of patients with a young child (Model 3), self-interest also appears to be a relevant predictor. While the probability of support reaches 44.2 per cent for respondents who have a young child in their household, this likelihood decreases by 6 percentage points for respondents without a young child (p < 0.001). This finding supports the self-interest hypothesis. The model also shows that younger respondents are more often in favor of prioritizing patients with a young child. On average, the probability of support increases by 0.2 percentage points with every additional year of age (p < 0.001). Furthermore, Model 3 indicates that women are less supportive of this criterion than men. The probability of support decreases by about 2.7 percentage points for female respondents compared to men (p < 0.001). Education also seems to influence attitudes towards the use of parental status as a criterion. While the probability for support is 36.2 per cent for respondents with a university education, it increases by 3.2 percentage points for respondents with an intermediate level of education and goes up to 41.1 per cent for respondents with a low level of education (p < 0.001). Moreover, the analysis shows that respondents in paid work are less in favor of the reference to parental status as a prioritization criterion than all other social positions. Hence, the hypothesis that unemployed people are significantly less in favor of this criterion than respondents in paid work must be rejected. In addition, for respondents in education, the effect is only significant at the 0.1 level. Although almost all model predictors seem to influence attitudes towards an explicit prioritization of patients with a young child, they only explain 1.1 per cent of the variance in the respondents’ positions.

## Discussion

Several recent studies have suggested that medical prioritization criteria are broadly accepted in society [26, 28]. However, experiences with prioritization in different countries indicate that rationing health services purely based on medical criteria cannot compensate for the financial challenges experienced by Western health care systems. Therefore, the purpose of this study was to analyze citizen attitudes towards the use of person-based characteristics as criteria for prioritization in health treatments if choices between patients with the same medical needs have to be made.

Although descriptive analysis indicates that the overall majority of the public, averaged over 28 nations, does not support the implementation of the analyzed indicators for person-based priority criteria, a substantial fraction would accept smoking habits, age and parental status as explicit health care priorities whenever it is impossible to treat everybody with the same medical need. Support is higher for the prioritization of younger patients (46 per cent) and non-smokers (42 per cent).

With regard to attitudes towards the use of patients’ smoking habits and parental status, self-interest seems to be the most relevant factor. This finding is in line with rational choice theory, which assumes that people tend to prefer the distribution mechanism that is the most advantageous for themselves. In contrast, respondents above the age of 70 support a prioritization of younger patients significantly more frequently than younger survey participants, which may rather indicate a preference for the fair-innings argument than for rational choice. Since women support all criteria significantly less often than men, gender also seems to be a relevant predictor for attitudes towards person-based priority criteria. Women thus seem to prefer health resource allocations based on need more often than men [28, 47]. This finding is in line with the hypothesis about differences in socialization between women and men [53]. Contrary to previous findings, the age of respondents does not seem to be related to respondents’ attitudes towards patients’ smoking habits [19, 28]. Moreover, our analysis shows that older respondents are significantly more likely to prioritize patients with a young child. Education only appears to have a significant influence on peoples’ attitudes towards the use of parental status as a priority criterion. Respondents with low or moderate education are significantly more in favor of a priority treatment for patients with young children than respondents with higher education.

It is important to also highlight the limitations of this study. First, all three criteria can be supported and rejected on grounds of two different distribution principles. While support for prioritization according to patients’ smoking habits as well as parental status could be an expression of efficiency or equality, the acceptance of age may express efficiency and fair innings motives. In contrast, the rejection of each criterion can be driven by preferences for distribution according to need or equity. Therefore, it is impossible to determine the underlying distribution principle for respondents’ support or rejection. Second, it is not clear that respondents would have the same attitude when two patients would be competing for a treatment other than heart surgery. Hence, the findings only apply for the scenarios used in the survey and cannot be transferred to other contexts. Third, the findings only apply for the three examined indicators representing the three discussed person-based priority criteria “health related lifestyle”, “age” and “social responsibility”. As a result, claims about attitudes towards other indicators, for example alcohol or drug consumption, cannot be made. Health related lifestyles as well as one’s social responsibility are complex realities. The indicators “patient’s smoking habit” and “being parent of a young child” embody only a small part of those personal aspects and therefore cannot be generalized towards conclusions about citizen attitudes with respect to priority setting based on lifestyle and social responsibility. Hence, in order to better understand attitudes towards the use of person based priority criteria, a broader range of indicators should be analyzed. Fourth, many individuals do not have the desire or capability to reach logical consistency across their preferences and moral beliefs. Instead, scenarios in which individuals are faced with a moral dilemma, like the question on how to distribute scarce resources, are usually influenced by their emotions and the social context [65]. As a consequence, the findings presented here may be driven more by the emotional state of the interviewed persons than by their moral beliefs and therefore might vary depending on affective content during the survey. These limitations suggest that our results should be interpreted with caution.

## Conclusion

According to the ISSP data studied in the present analysis, a majority of citizens across most countries reject age, lifestyle, and social responsibility as prioritization criteria in the allocation of health treatments. Our analysis also suggests that the acceptance probability for all three criteria is, to different extents, related to self-interest, age, gender, level of education, and social position. We conclude by raising two concerns. Even if a majority of citizens were to favor prioritization with respect to health care, this does not alleviate the ethical problem. Decisions on how to allocate scarce resources are embedded in moral choices about who should receive treatment and who should be excluded [66]. Those choices unavoidably include moral judgments and therefore present a distinctive ethical challenge [65]. This concern is particularly acute if prioritization rules correlate with social or ascriptive criteria, such as race or gender. Priority criteria, therefore, need to be defensible in ethical terms before they can potentially be considered policy relevant [67].

The second concern is methodological. Public attitudes towards the use of person-based priority criteria vary between countries but only a small share of the variance is directly explained by predictors at the country level. Our individual-level cross-country analysis demonstrates that several individual factors affect attitudes towards person-based criteria for prioritization rules. Nevertheless, the variance explained by these individual predictors is very low as well. The findings also suggest that individual predictors do not have the same influence in every country. Thus, the variation in support does not directly depend on differences between the countries or variation across respondents’ self-interest and social conditions. A promising avenue for further research may be to explore the ways in which country characteristics moderate the effects of individual-level predictors of attitudes towards prioritization in health care. Future research should thus focus on identifying cross-level conditionalities between country and individual characteristics.

## Supporting Information

S1 Table Fixed-Effect logistic regression models: influencing factors on peoples’ support of smoking habits, age and parenthood of a minor child as priority criteria. (DOCX)
