# Supplementary material for: Who Shall Not Be Treated: Public Attitudes on Setting Health Care Priorities by Person-Based Criteria in 28 Nations
Source: PLoS One. 2016 Jun 9;11(6):e0157018. doi: 10.1371/journal.pone.0157018 (PMC4900563; doi:10.1371/journal.pone.0157018)
Supplement: S1 Table — (DOCX) [file pone.0157018.s001.docx]

**S1 Table: Fixed-Effect logistic regression models: influencing factors on peoples’ support of smoking habits, age and parenthood of a minor child as priority criteria**

|  | M1: Support for smoking habits | M2: Support for age | M3: Support for parenthood of a minor child |
| --- | --- | --- | --- |
| Smoker | -0.989^***^ |  |  |
|  | (-32.68) |  |  |
| Age 70 or older |  | 0.253^***^ |  |
|  |  | (6.26) |  |
| Young child in household |  |  | 0.256^***^ |
|  |  |  | (7.94) |
| Age in years | -0.000 | - | 0.009^***^ |
|  | (-0.34) |  | (8.94) |
| Female | -0.063^**^ | -0.082^***^ | -0.111^***^ |
|  | (-2.69) | (-3.70) | (-4.84) |
| Level of education (ref. high) |  |  |  |
| Low | -0.036 | 0.029 | 0.206^***^ |
|  | (-1.12) | (0.96) | (6.42) |
| Intermediate | -0.021 | 0.028 | 0.136^***^ |
|  | (-0.72) | (1.01) | (4.62) |
| Social position (ref. in paid work) |  |  |  |
| Unemployed | -0.074 | 0.093 | 0.196^***^ |
|  | (-1.46) | (1.96) | (4.02) |
| In school | 0.169^**^ | 0.199^***^ | 0.217^***^ |
|  | (3.10) | (4.08) | (3.92) |
| In training/student | 0.297 | 0.269 | 0.308 |
|  | (1.77) | (1.68) | (1.81) |
| Permanently sick/disabled | -0.031 | -0.037 | 0.203^**^ |
|  | (-0.46) | (-0.58) | (3.12) |
| Retired | 0.011 | -0.142^***^ | 0.219^***^ |
|  | (0.29) | (-4.23) | (5.74) |
| Others | 0.079 | 0.147^***^ | 0.314^***^ |
|  | (1.86) | (3.57) | (7.42) |
| Household income in 1000 international $ | 0.000 | 0.000 | 0.000 |
|  | (0.62) | (1.79) | (-0.04) |
| Constant | 0.845^***^ | 0.086 | -0.839^***^ |
|  | (10.04) | (1.28) | (-10.17) |
| *N* | 36199 | 36199 | 36199 |
| Average adjusted R2 (McFadden) | 0.072 | 0.023 | 0.050 |
| Average adjusted R2 (McFadden) fixed effects only | 0.045 | 0.021 | 0.039 |

Coefficients are Logits; t statistics in parentheses; ^*^ p < 0.05, ^**^ p < 0.01, ^***^ p < 0.001; Multiple Imputation (m=5)
